# Variability in the production of tannins and other polyphenols in cell cultures of 12 Nordic plant species

**DOI:** 10.1007/s00425-017-2686-8

**Published:** 2017-04-05

**Authors:** Jussi Suvanto, Liisa Nohynek, Tuulikki Seppänen-Laakso, Heiko Rischer, Juha-Pekka Salminen, Riitta Puupponen-Pimiä

**Affiliations:** 1 Natural Chemistry Research Group, Department of Chemistry, University of Turku, 20014 Turku, Finland; 2 VTT Technical Research Centre of Finland Ltd., 02044 Espoo, Finland

**Keywords:** Anthocyanins, Flavonoids, Hydrolysable tannins, Plant cell suspension, Proanthocyanidins, UHPLC-DAD–MS^2^

## Abstract

**The polyphenol profiles of 18 cell cultures from 12 plant species were screened. The detected polyphenol fingerprints were diverse and differed from polyphenol profiles typically found in corresponding plant species.**

Cell cultures originating from 12 different plant species growing or grown in the Nordic countries were screened for their ability to synthesize polyphenols to assess their suitability for future studies and applications. The focus was on plant families Rosaceae and Ericaceae. On average, the Rosaceae cultures were the most efficient to produce hydrolysable tannins and the Ericaceae cultures were the most efficient to produce proanthocyanidins. This is in line with the general trend of polyphenols found in Rosaceae and Ericaceae leaves and fruits, even though several individual cell cultures differed from natural plants in their polyphenolic composition. Overall, several of the studied cell cultures exhibited capability in producing a large variety of polyphenols, including tannins with a high molecular weight, thus also showing promise for further studies concerning, for example, the accumulation of specific polyphenols or biosynthesis of polyphenols in the cell cultures.

## Introduction

Polyphenols are a diverse group of plant secondary metabolites found throughout the plant kingdom, encompassing such subgroups as tannins and flavonoids, among others. In plants, their biological function lies mostly within their defensive capabilities against herbivores, pathogens, and UV-B radiation (Haukioja 1991). In addition, numerous polyphenolic compounds have been found to be bioactive, for instance, due to their antioxidative, antimicrobial, antiviral, and antitumor activities (Okuda et al. 2009; Serrano et al. 2009). Tannins form structurally the most complex group of polyphenols, and they can be divided into hydrolysable tannins, proanthocyanidins (syn. condensed tannins), and phlorotannins. Plants rich in tannins have traditionally found use in leather tanning, hence their name, resulting from their ability to bind and precipitate proteins.

Publications reporting polyphenols from callus cultures date back to at least the 1960s (e.g., Constabel 1968; Davies 1972), and different polyphenolic compound groups identified include galloyl glucoses (e.g. Haddock et al. 1982), gallotannins (e.g., Krajci and Gross 1986), ellagitannins (e.g., Scalbert et al. 1988), proanthocyanidins (e.g., Oberthur et al. 1983), and anthocyanins (e.g., Nawa et al. 1993), and other flavonoids (e.g., Oberthur et al. 1983). The use of plant cell cultures, in general, could be beneficial in polyphenol studies, as they grow under very strictly and accurately controlled conditions allowing easy addition of hormones, biosynthetic precursors, and other compounds of interest. It has also been recently shown with *Rubus chamaemorus* that suspension cultures of plant cells can be scaled up to an industrial scale to create cell material having a defined chemical, including polyphenolic composition (Nohynek et al. 2014). This kind of mass-produced plant cell material can be used in various fields of industry, such as food and cosmetics, in place of natural plants to ensure consistent quality and to minimize the dependency on natural crops of plants that can vary both in quality and availability. Furthermore, for endangered species, biotechnical means of growing plant material in cell cultures may be the only way especially for industrial scale utilization in a sustainable way in the future, as has been suggested in some recent publications (Matkowski 2008; Nohynek et al. 2014).

Altogether, several of the aforementioned bioactivities are beneficial when tannins or other polyphenols, plants rich in them or cell cultures derived from polyphenol-rich plants are used in cosmetic (Barbulova et al. 2014; Zillich et al. 2015) and nutraceutical (Espín et al. 2007) products. In the cosmetic applications, the usage of polyphenols is often related to their purported anti-aging properties, caused mainly by their protection against lipid oxidation (Hagerman et al. 1998), ability to inhibit, e.g., tyrosinase activity on the skin (Parvez et al. 2007) and stabilize collagen (Madhan et al. 2005), and their photoprotective capabilities against UV radiation (Nichols and Katiyar 2010).

For this study, a total of 18 cell suspension cultures originating from 12 plant species, of which 10 were originally from Finland and one from Sweden and Norway each, were chosen for analyses on their polyphenolic profile by UHPLC–MS^2^ (ultra-high-performance liquid chromatography–tandem mass spectrometry). This screening was done to see which cultures would provide suitable platforms for future studies on producing polyphenols in a laboratory environment. The plant species represented eight different genera (*Rubus*, *Fragaria*, *Sorbus*, *Vaccinium*, *Empetrum*, *Lonicera*, *Avena*, and *Hordeum*) and four families (Rosaceae, Ericaceae, Caprifoliaceae, and Poaceae). For most of the species included, the previous cell culture studies concerning polyphenolic compounds are non-existent.

## Materials and methods

### Chemicals

ACS grade acetone was from VWR International S.A.S. (Fontenay-sous-Bois, France). LC–MS grade acetonitrile and formic acid was from Fluka Analytical (Sigma Aldrich Chemie GmbH, Steinheim, Germany). Water was purified with Millipore Synergy UV (Merck KGaA, Darmstadt, Germany) water purification system.

### Cell cultures

Most of the used plant cell lines (Table 1) are included in VTT Technical Research Centre of Finland’s proprietary culture collection (http://culturecollection.vtt.fi/) cryo-stored in liquid nitrogen or maintained as callus culture on solid medium by sub-culturing the cells regularly. The plant species included cloudberry (*R. chamaemorus* L., sample codes Rc1 and Rc2), raspberry (*Rubus idaeus* L., sample code Ri), arctic bramble (*Rubus arcticus* L., sample codes Ra1 and Ra2), stone bramble (*Rubus saxatilis* L., sample codes Rs1 and Rs2), strawberry (*Fragaria* × *ananassa* (*Duchesne ex Weston*) *Duschesne ex Rozier ‘Senga Sengana’*, sample code Fa), mountain ash (*Sorbus aucuparia* L., sample codes Sa1 and Sa2), bilberry (*Vaccinium myrtillus* L., sample codes Vm1 and Vm2), lingonberry (*Vaccinium vitis*-*idaea* L., sample code Vv), black crowberry (*Empetrum nigrum* L., sample code En), blue honeysuckle (*Lonicera caerulea* L. var. *kamtschatica*, sample code Lc), oat (*Avena sativa* L., sample code As), and barley (*Hordeum vulgare* L., sample codes Hv1 and Hv2). *R. saxatilis* was originally from Nesland, Lofoten, Norway, and *L. caerulea* from Överkalix, Sweden. The rest were from Finland.

**Table 1:** Studied plant cell cultures, their VTT culture collection codes, colours of the culture, and used growth media

Sample code	Plant cell culture	VTT culture collection code	Colour of the culture	Growth medium^b^
Rc1	*R. chamaemorus* L.	VTT P-120083	Yellow	MS
Rc2	*R. chamaemorus* L.	VTT P-120083	Yellow	MS
Ri	*R. idaeus* L.	VTT P-120090	Green	MS
Ra1	*R. arcticus* L.	VTT P-120089	Red	MS-pH4
Ra2	*R. arcticus* L.	VTT P-120087	Green	MS-pH4
Rs1	*R. saxatilis* L.	VTT P-120091	Green	MS
Rs2	*R. saxatilis* L.	VTT P-120091	Yellow	MS
Fa	*Fragaria* × *ananassa* (*Duchesne ex Weston*) *Duschesne ex Rozier ‘Senga Sengana’*	VTT P-120010	Green	MS-pH4
Sa1	*S. aucuparia* L.	VTT P-120084	Green	MS
Sa2	*S. aucuparia* L.	VTT P-120084	Green	MS
Vm1	*V. myrtillus* L.	VTT P-120045	Brownish	WP
Vm2	*V. myrtillus* L.	VTT P-120045	Dark red	WP
Vv	*V. vitis*-*idaea* L.	KAS 377^a^	Yellow	WP
En	*E. nigrum* L.	KAS 446^a^	Dark red	WP
Lc	*L. caerulea* L. var. *kamtschatica*	KAS 469^a^	Yellow	MS
As	*A. sativa* L. ‘Veli’	VTT P-120073	Yellow	MS-mod
Hv1	*H. vulgare* L. ‘Pokko’	VTT EC-P-120080	Yellow	B5-mod
Hv2	*H. vulgare* L. ‘Wolmari’	VTT P-120075	Yellow	CIM

^a^No VTT culture collection code available, from the plant collection of Plant Biotechnology Team at VTT

^b^ *MS* Murashige and Skoog medium (Murashige and Skoog 1962) (Duchefa Biochemie, The Netherlands) containing 3% (w/v) sucrose, 0.1 mg l^−1^ kinetin (Sigma), and 1 mg l^−1^ α-naphthaleneacetic acid (Sigma). For solid medium, 8 g l^−1^ Bacto agar (BD, Becton, Dickinson and Company, USA) was added; MS-pH 4 = As MS, but pH was decreased to 4.0 before autoclaving; *WP* woody plant medium (Duchefa Biochemie) containing 3% (w/v) sucrose, 2.2 mg l^−1^ thidiazuron (Sigma P6186), and 1.95 mg l^−1^ α-naphthaleneacetic acid. For solid medium, 8 g l^−1^ Bacto agar was added; *MS-mod* modified MS medium (Ritala et al. 2007) containing 2% (w/v) sucrose, 150 mg l^−1^ asparagine, and 2.0 mg l^−1^ 2,4-dichlorophenoxyacetic acid (Sigma). For solid medium, 3 g l^−1^ Phytagel (Sigma) was added; *B5-mod* modified B5 medium (Ritala et al. 1993) containing 3% (w/v) sucrose and 4.0 mg l^−1^ 2,4-dichlorophenoxyacetic acid. For solid medium, 2 g l^−1^ Phytagel was added; *CIM* medium as described by Ritala et al. (2008)

Suspension cultures of plant cell lines were initiated from soft callus, and cultivated in medium optimal for each cell line (Table 1) at 24 ± 1 °C on an orbital shaker at 100–110 rpm. The plant cell lines As and Hv1 were cultivated in dark, the rest under a day–night illumination regime (photoperiod 16:8 h, irradiation 40 µmol m^−2^ s^−1^). The cultures were sub-cultured regularly in fresh medium every 10 ± 4 days, depending on cell line, and step-wise up-scaled to 250 ml flasks containing 60 ml of culture. When sub-cultured, the biomass was diluted 1:3 and clumps of the callus were removed.

In general, the plant cells were cultivated in Erlenmeyer flasks from 70 to 700 ml as culture volume at the conditions described above for each cell line. As exceptions, *R. chamaemorus* cells (Rc1 and Rc2) were obtained from two subsequent large scale cultivations in a fermenter of 300 l (Nohynek et al. 2014), and *S. aucuparia* cells (Sa1 and Sa2) were produced in BIOSTAT^®^ CultiBag RM Wave bioreactor, consisting of a rocking unit (BioWave 20SPS; Wave Biotech AG, Tagelswangen, Switzerland) and a cultivation bag (CultiBag; Sartorius AG, Göttingen, Germany) of 2 l. The plant cells were separated from the medium by vacuum filtration using a Büchner funnel and Miracloth tissue (Calbiochem, Merck KGaA), washed twice with sterile MilliQ water, and lyophilized. The lyophilized cell biomass was kept frozen (−20 °C) until extraction.

### Extraction

20 mg of lyophilized and ground sample material was macerated overnight in 1400 µl of acetone/water (4:1, v/v) in a 2 ml Eppendorf tube at 4 °C, extracted on a planar shaker for 3 h, and centrifuged, after which the supernatant was recovered. The extraction was repeated (omitting the maceration) and the supernatants were combined. Acetone was evaporated from the extracts in vacuo, and the extracts were finally frozen, lyophilized, and stored in −20 °C until analyses. These extracts were used to determine all compounds except for anthocyanins. The total anthocyanin content was determined from methanol extracts of lyophilized and powdered samples showing at least traces of red, blue, or purple colour, as evaluated by eye. 10 mg of the sample was mixed with 500 µl of methanol in an Eppendorf tube, vortexed vigorously for 2 min and after 15 min of standing and occasional mixing centrifuged at 7800×*g* for 5 min. The supernatant was separated and stored in a glass LC vial at −20 °C until analysis.

### UHPLC-DAD–3Q–MS^2^

The first U(H)PLC system (Acquity UPLC^®^, Waters Corporation, Milford, MA, USA) consisted of a binary solvent manager, a sample manager, a column (Acquity UPLC BEH Phenyl, 1.7 µm, 2.1 × 100 mm, Waters Corporation), and a photodiode array detector (DAD). The photodiode array detector was set to operate between 190–500 nm. The UHPLC system was connected to a Waters triple quadrupole mass spectrometer (3Q-MS^2^; Xevo TQ, Waters Corporation) using electrospray ionization (ESI).

Lyophilized extracts were dissolved in 200 µl of water and filtered using a syringe filter (0.2 µm PTFE, 13 mm, VWR International GmbH, Darmstadt, Germany). Sample injection volume was 5 µl. 0.1% formic acid (A) and acetonitrile (B) were used as eluents and the flow rate was kept at 0.5 ml min^−1^ with the gradient as follows: 0.0–0.5 min, 0.1% B in A; 0.5–5.0 min, 0.1–30.0% B in A (linear gradient); 5.0–8.0 min, 30.0–45.0% B in A (linear gradient); column wash and stabilization.

Negative full scan mode was used with ions being detected between *m/z* (mass-to-charge ratio) 150–1200. Group-specific multiple reaction monitoring methods used for the detection of ellagitannins, gallic acid derivatives, quinic acid derivatives, kaempferol derivatives, quercetin derivatives, myricetin derivatives, and procyanidin and prodelphinidin terminal, and extensions units were as described by Engström et al. (2014, 2015). External calibration was used and the calibration curves were done by using purified tellimagrandin I (0.47–7.50 µg ml^−1^) for ellagitannins, pentagalloyl glucose (0.06–1.00 µg ml^−1^) for gallic acid derivatives, chlorogenic acid (0.16–2.50 µg ml^−1^) for quinic acid derivatives, kaempferol-3-*O*-glucoside (0.13–2.00 µg ml^−1^) for kaempferol derivatives, quercetin-3-*O*-galactoside (0.13–2.00 µg ml^−1^) for quercetin derivatives, myricetin-3-*O-*rhamnoside (0.56–9.00 µg ml^−1^) for myricetin derivatives, and procyanidin (59–434 µg ml^−1^) and prodelphinidin (159–1112 µg ml^−1^) rich Sephadex LH-20 purified fractions for procyanidins and prodelphinidins. An ESI source was used with the temperature set at 150 °C, desolvation temperature at 650 °C, and capillary voltage at 3.4 kV. For full scan, a cone voltage ramp (30 V at *m/*z 200 to 50 V at *m/z* 1000) was used. N_2_ was used as desolvation gas (1000 l h^−1^) and cone gas (100 l h^−1^). Argon was used as collision gas. The quantitation was done using the TargetLynx software (V 4.1, Waters Corporation).

### Anthocyanin analyses

A second UHPLC system (Waters Acquity UPLC H-Class, Waters Corporation) equipped with an Acquity UPLC BEH C18 column (1.7 µm, 2.1 × 100 mm, Waters Corporation) was used for the analysis of anthocyanins. Used eluents were 0.1% formic acid (A) and acetonitrile (B), sample injection volume was 3 µl, solvent flow rate 0.3 ml min^−1^, and the gradient as follows: 0.0–1.1 min, 5–10% B in A (linear gradient); 1.1–5.7 min, 10–60% B in A (linear gradient); 5.7–9.0 min, 60–90% B in A (linear gradient); 9.0–11.0 min, 90–100% B in A (linear gradient); column wash and stabilization. Quantification of anthocyanins was done by UV detection at 520 nm. Cyanidin-3-glucoside was used as an external standard and the calibration curve was determined at a concentration range of 0.59–63.83 µg ml^−1^.

The identification on anthocyanins was confirmed on a Waters Q-Tof Premier mass spectrometer coupled to a Waters Acquity UPLC using the same column and gradient as for anthocyanin quantification. Electrospray ionization was used in positive mode and ions were detected between *m/z* 100–1200. The conditions were set at as follows: capillary voltage 3.0 kV, sample cone voltage 45 V, ion source temperature 120 °C, desolvation gas (N_2_) flow and temperature 800 l h^−1^ and 270 °C, and collision gas (Ar) flow 0.60 l h^−1^. Reserpine was used as a lock spray compound.

### UHPLC-DAD–Orbitrap–MS^2^

A third UHPLC–MS system was used to achieve accurate mass spectral data on samples that showed the most promising polyphenolic fingerprints (samples Sa1, Vm1, and En). The UHPLC system, including the column, was identical to the one in the first system, but it was connected to a hybrid quadrupole-Orbitrap mass spectrometer (Q Exactive™, Thermo Fisher Scientific GmbH, Bremen, Germany).

The extracts made for UHPLC-DAD–3Q-MS^2^ analyses from these three samples were used as is for UHPLC-DAD–Orbitrap-MS^2^ analyses. Sample injection volume was 5 µl. 0.1% formic acid (A) and acetonitrile (B) were used as eluents and the flow rate was 0.5 ml min^−1^. The used gradient was as follows: 0.0–0.5 min, 0.1% B in A; 0.5–5.0 min, 0.1–30.0% B in A (linear gradient); 5.0–8.0 min, 30.0–45.0% B in A (linear gradient); column wash and stabilization.

A heated ESI source (H-ESI II, Thermo Fisher Scientific GmbH) was operated in negative ion mode and the following parameters were used: spray voltage, −3.0 kV; sheath gas (N_2_) flow rate, 60 (arbitrary units); aux gas (N_2_) flow rate, 20 (arbitrary units); sweep gas flow rate, 0 (arbitrary units); capillary temperature, 380 °C. A resolution of 70,000 was used in the Orbitrap detector, an automatic gain of 3 × 10^6^ was used, and the mass range was set at *m/z* 150–2000. For compound fragmentation studies, full MS/dd-MS^2^ (TopN) experiments were performed using a loop count and TopN value of 5, a resolution of 17,500, and an automatic gain of 1 × 10^5^. Pierce ESI Negative Ion Calibration Solution (Thermo Fischer Scientific Inc., Waltham, MA, USA) was used for the calibration of the detector. The data were processed with the Thermo Xcalibur Qual Browser software (Version 3.0.63, Thermo Fisher Scientific Inc.).

## Results and discussion

Gallic acid derivatives, ellagitannins, quinic acid derivatives, kaempferol derivatives, quercetin derivatives, myricetin derivatives, procyanidins, and prodelphinidins were quantified from all the 18 cell cultures using the UHPLC–MS^2^ methods described earlier by Engström et al. (2014, 2015). The concentrations of each of the quantified polyphenol groups are presented in Table 2. All plant cell cultures except for *R. idaeus* contained at least trace amounts of procyanidins and gallic acid derivatives, the latter of which consisted mainly of monogalloyl glucose isomers in all samples. Suspension cultures from three species, *S. aucuparia*, *V. myrtillus*, and *E. nigrum*, proved exceptionally interesting in their polyphenol content both qualitatively and quantitatively. One culture from all three of these species was chosen for more accurate qualitative analyses by Orbitrap mass spectrometry.

**Table 2:** Concentrations of the different polyphenolic compound groups in each of the suspension culture samples

Sample code	Concentration (mg g^−1^ dry weight)								
	Gallic acid derivatives	Ellagitannins	Quinic acid derivatives	Kaempferol derivatives	Quercetin derivatives	Myricetin derivatives	Procyanidins	Prodelphinidins	Anthocyanins
Rc1	tr	–	–	–	–	–	–	–	na
Rc2	0.07	–	–	–	–	–	0.27	–	na
Ri	–	–	–	–	–	–	–	–	na
Ra1	tr	–	–	0.23	0.44	tr	0.18	–	4.0
Ra2	tr	–	–	–	–	tr	tr	–	na
Rs1	0.11	tr	–	–	–	–	tr	–	na
Rs2	0.15	tr	–	–	–	–	tr	–	–
Fa	tr	–	–	–	–	–	0.07	–	na
Sa1	0.33	tr	–	–	–	–	tr	–	na
Sa2	1.02	0.60	–	–	–	–	tr	–	–
Vm1	tr	–	–	0.06	0.18	–	20.88	0.17	2.8
Vm2	tr	–	–	0.08	0.21	–	26.26	0.18	4.8
Vv	tr	–	–	–	0.08	–	0.22	–	–
En	tr	–	–	0.08	tr	–	3.04	2.03	3.8
Lc	tr	–	0.60	–	–	–	tr	–	na
As	tr	–	–	–	–	–	tr	–	na
Hv1	tr	–	–	–	–	–	tr	–	na
Hv2	tr	–	tr	–	–	–	tr	–	–

*tr* trace amounts, *na* not analyzed

### Family Rosaceae

A total of 10 cell suspension cultures were included from the family Rosaceae, representing three genera (*Rubus*, *Fragaria*, and *Sorbus*). From the genus *Rubus,* a total of seven cultures from four species (*R. chamaemorus* L., *R. idaeus* L., *R. arcticus* L., and *R. saxatilis* L.) were included*. Rubus* plants are most often characterized by high ellagitannin concentration in both their berries (Kähkönen et al. 2001; Nohynek et al. 2006) and leaves (Okuda et al. 1992), with the main ellagitannins typically being the dimeric sanguiin H-6 and the trimeric lambertianin C. Other phenolic compounds in *R. idaeus* include anthocyanins in fruits and flavonols, flavan-3-ols, proanthocyanidins, ellagic acid conjugates, and phenolic acids in both fruits and leaves (Harborne and Hall 1964; Ryan and Coffin 1971; Henning 1981; Törrönen et al. 1997; Kähkönen et al. 2001; Määttä-Riihinen et al. 2004b). Both fruits and leaves of the other three *Rubus* species included in the study are known to include very similar compounds to *R. idaeus* (e.g., Okuda et al. 1992; Törrönen et al. 1997; Häkkinen et al. 1999; Kähkönen et al. 2001; Määttä-Riihinen et al. 2004b).

In contrast, from the cultures included in this study, only the ones originating from *R*. *saxatilis* contained detectable levels of ellagitannins. In addition, the ellagitannins observed were determined to be monomers, and no traces of the characteristic oligomeric ellagitannins were detected. Besides ellagitannins, gallic acid derivatives were detected in all *Rubus* cultures except *R. idaeus*. Small amounts of proanthocyanidins were detected in five out of seven of the *Rubus* cultures, and flavonoids only in *R. arcticus* cultures. Anthocyanins were detected in Ra1 as cyanidin, delphinidin, petunidin, and peonidin glucosides, galactosides, and/or arabinosides.

*Fragaria* × *ananassa* (Duchesne ex Weston) Duschesne ex Rozier ‘Senga Sengana’ was represented by one culture, and it proved to be fairly similar to several of the *Rubus* cultures in that it mainly consisted of small amounts of procyanidins and trace quantities of gallic acid derivatives. Earlier, López Arnaldos et al. (2001) have studied the changes in total soluble phenolics and flavanols, (+)-catechin, and ferulic acid and its glucoside in *F*. × *ananassa* callus cultures during their growth. They found that the concentrations of phenolic compounds peaked in the beginning of the exponential growth phase. As can be noted, the compounds they detected were not in line with the compound groups we identified to be present in our *F*. × *ananassa* suspension culture, which is most likely explained by different culture conditions, age, heritage of the culture, and different cultivar.

The phenolic content of the fruits of *F.* × *ananassa* has been studied fairly thoroughly, and they are known to contain ellagitannins and ellagic acid derivatives, proanthocyanidins, anthocyanins and other flavonoids including flavonols and flavan-3-ols, and hydroxycinnamic acids (Gil et al. 1997; Häkkinen et al. 1999; Aaby et al. 2007; Buendía et al. 2010).

The leaves of *F.* × *ananassa*, on the other hand, are not nearly as studied as the fruits, but they are known to contain ellagitannins, ellagic acid derivatives, galloyl glucoses, proanthocyanidins, flavonoids, and hydroxycinnamic acids (Skupień and Oszmiański 2004; Kårlund et al. 2014).

**Fig. 1:**
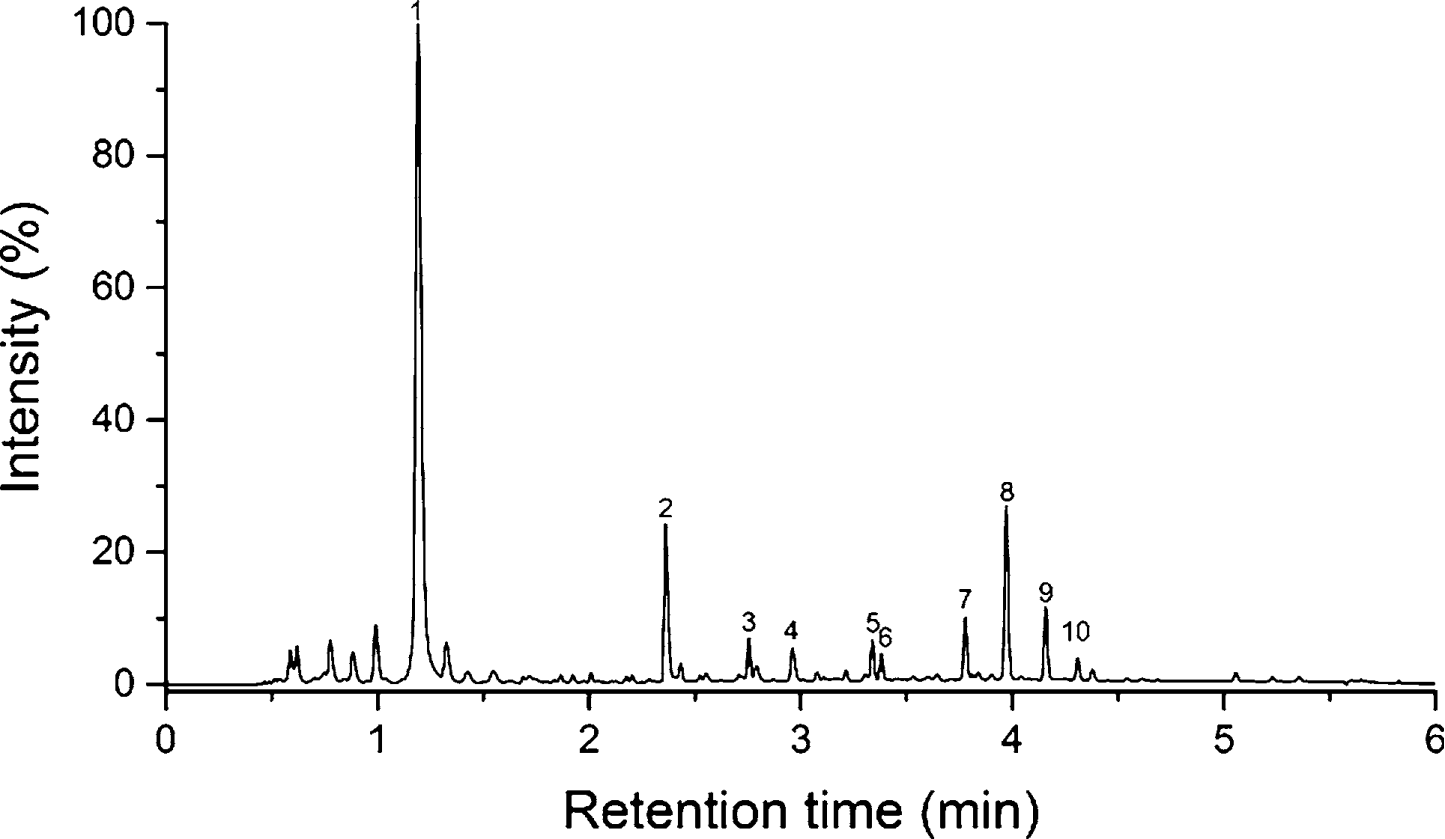
UHPLC-DAD chromatogram (*λ* = 280 nm) of the *S. aucuparia* suspension culture sample Sa2. For peak identification, see Table 3

**Fig. 2:**
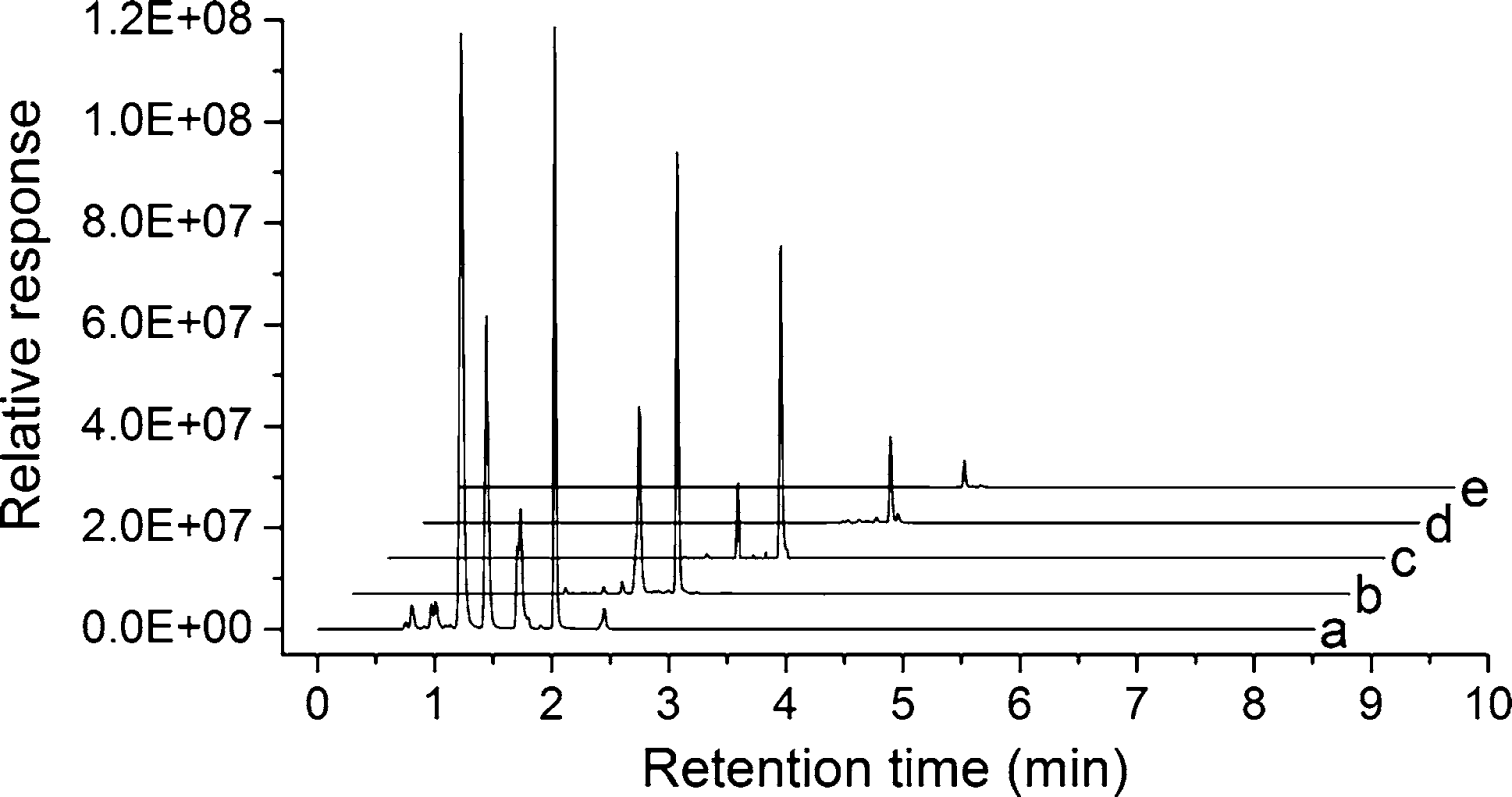
Extracted ion chromatograms from *S. aucuparia* corresponding to the *m/z* values of mono- (*a*), di- (*b*), tri- (*c*), tetra- (*d*), and pentagalloyl (*e*) glucoses

Two *S. aucuparia* L. suspension cultures cultivated by different methods were included. The culture Sa1 was cultivated in shake flasks, whereas the culture Sa2 was grown in a plastic cultivation bag in a wave type bioreactor. Their phenolic profiles were similar, consisting mostly of galloyl glucoses, ellagitannins, and procyanidins. Quantitatively culture Sa2, which was grown in a wave type bioreactor, had higher concentrations of both gallic acid derivatives and ellagitannins, showing its higher potential to produce these successive compound groups of the hydrolysable tannin pathway. The culture grown in shake flasks, Sa1, produced ellagitannins in trace quantities only, as did both *S. aucuparia* cultures procyanidins. The UV chromatogram (*λ* = 280 nm) of the *S. aucuparia* culture Sa2 is presented in Fig. 1 and a more detailed characterization of the compounds detected in it is presented in Table 3. The main ellagitannins were identified as two isomeric galloyl-bis-HHDP-β-d-glucopyranoses and one trigalloyl-HHDP-β-d-glucopyranose. Galloyl glucoses from monogalloyl glucose to di-, tri-, tetra-, and pentagalloyl glucoses were detected as well (Fig. 2), with mono- to trigalloyl glucoses appearing as several isomers.

**Table 3:** Compound identification for the *Sorbus aucuparia* culture sample Sa2

Peak	Tentative compound identification	Molecular formula	MS^1^ (*m/z*)	MS^2^, characteristic fragments (*m/z*)^a^	Exact mass, observed (g mol^−1^)	Exact mass, calculated (g mol^−1^)	Error (ppm)
1	1-*O*-Monogalloyl-β-d-glucose	C_13_H_16_O_10_	331.0670 [M−H]^−^	(331) 169.0130, 125.0227	332.0737	332.0743	−1.8
2	Unknown		407.1722 203.0818	(203) 159.0914, 142.0647, 116.0489, 72.0072			
3	Digalloyl glucose	C_20_H_20_O_14_	483.0783 [M−H]^−^	(483) 313.0565, 169.0129	484.0850	484.0853	−0.6
4	Unknown		431.1193	(431) 223.0606, 208.0369, 179.0703, 164.0466			
			385.1141	(385) 223.0604, 208.0368, 179.0701, 164.0465			
5	Trigalloyl glucose	C_27_H_24_O_18_	635.0895 [M−H]^−^	(635) 483.0778, 465.0672, 169.0129	636.0962	636.0963	−0.2
6	Unknown		771.2357 385.1141	(771) 223.0604, 208.0367, 179.0701, 164.0464			
7	Galloyl-bis-HHDP-β-d-glucopyranose	C_41_H_28_O_26_	935.0805 [M−H]^−^ 467.0362 [M−2H]^2−^	(467) 300.9986, 275.0193, 169.0128	936.0872	936.0869	0.4
8	Trigalloyl-HHDP-β-d-glucopyranose	C_41_H_30_O_26_	937.0956 [M−H]^−^ 468.0438 [M−2H]^2−^	(468) 300.9987, 275.0196, 249.0402, 169.0128, 125.0227	938.1023	938.1025	−0.2
9	Galloyl-bis-HHDP-β-d-glucopyranose	C_41_H_28_O_26_	935.0797 [M−H]^−^ 467.0361 [M−2H]^2−^	(467) 633.0732, 300.9986, 275.0195, 249.0400, 169.0129	936.0864	936.0869	−0.6
10	Pentagalloyl glucose	C_41_H_30_O_26_	939.1123 [M−H]^−^ 469.0521 [M−2H]^2−^	(939) 769.0902, 617.0784, 465.0670, 447.0567, 169.0129, 125.0228	940.1188	940.1182	0.7

Peak numbers correspond to those presented in Fig. 1

^a^The ions marked in parentheses were used for MS^2^ fragmentation experiments

The identities of these hydrolysable tannins were further confirmed by MS^2^ experiments. Galloyl glucoses showed characteristic fragments at *m/z* values 169 and 125, corresponding to gallic acid and a subsequent cleavage of its CO_2_ unit (Lee et al. 2005). Fragments resulting from the cleavage of a galloyl or a gallic acid unit were detected, resulting in a loss of 152 or 170 Da from the precursor ion, respectively. Ellagitannins were similarly detected by the presence of ions at *m/*z values 301, 275, and 249 corresponding to ellagic acid, and a further loss of either one or two CO_2_ units, and a corresponding loss of 302 Da from the precursor ion. The fragments presented for galloyl glucoses are often observed with ellagitannins, as they can also include galloyl groups. The identity of peak 2 could not be solved, but the accurate mass measurements suggest that it might contain two nitrogen atoms, making it not a true polyphenol, and that the two detected ions are [M−H]^−^ and [2M−H]^−^. Peaks 4 and 6 are very likely related to each other, as they have similar fragmentation patterns, and they might be hexosides, as the ion at *m/z* 223, resulting from a 162 Da loss from the likely [M−H]^−^ ion at *m/z* 385, was of high intensity.

The compounds identified are in clear contrast to *S*. *aucuparia* berries, the phenolic profile of which has been reported to consist of hydroxycinnamic acids, hydroxybenzoic acids, anthocyanins, procyanidins, flavonols, and flavanols (Kähkönen et al. 2001), with 3-caffeoylquinic acid being the main compound contributing to 46% of 18.83 mg g^−1^ DW of total phenolics (Kylli et al. 2010). The leaves and inflorescences of *S*. *aucuparia* have not been studied as widely as the berries, but they contain phenolic acids, proanthocyanidins, and flavonoids (Olszewska and Michel 2009).

The profiles of the studied Rosaceae species cultures shared some similarities. Their average gallic acid derivative concentrations were higher than in the cultures of other plant families, and ellagitannins were only detected in Rosaceae cultures, albeit only in two of the six included species. This is in line with the fact that plants in the family Rosaceae are generally rich sources of hydrolysable tannins (Moilanen et al. 2015), and the possibility of using oligomeric ellagitannins as chemotaxonomic markers in Rosaceae has been suggested by Okuda et al. (1992), for example. All of the galloyl glucose producing Rosaceae cell cultures contained 1-*O*-monogalloyl-β-d-glucose, which is biosynthetically the first hydrolysable tannin, a precursor of all other hydrolysable tannins, and, therefore, an important intermediate in the hydrolysable tannin pathway. It remains unknown why it was not efficiently converted to biosynthetically following hydrolysable tannins.

### Family Ericaceae

**Fig. 3:**
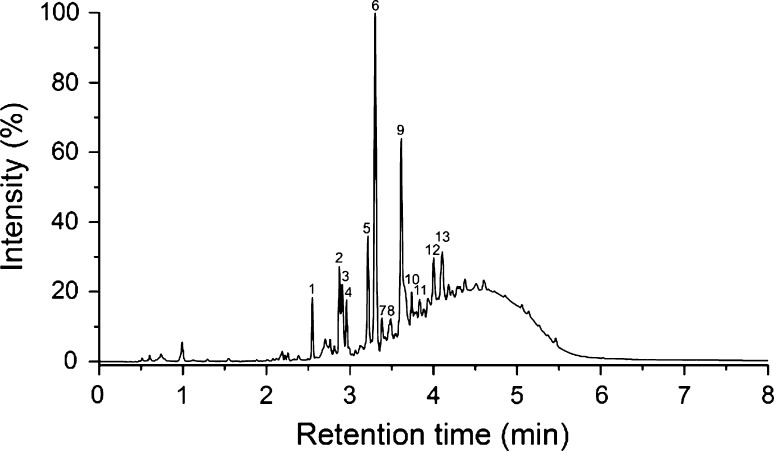
UHPLC-DAD chromatogram (*λ* = 280 nm) of the *V. myrtillus* suspension culture sample Vm2. For peak identification, see Table 4

A total of four cell suspension cultures were included from the family Ericaceae, encompassing two genera (*Vaccinium* and *Empetrum*) and three species. *V. myrtillus* L. proved to be the most efficient producer of procyanidins among all studied suspension cultures by far; two *V. myrtillus* cultures were studied, and they contained 20.88 mg g^−1^ DW and 26.26 mg g^−1^ DW of procyanidins, contributing to 98% of total polyphenols. Several A-type proanthocyanidins were detected, which is in line with what has been found in *V*. *myrtillus* in nature (Hokkanen et al. 2009). As for other proanthocyanidins, prodelphinidins were found in much smaller quantities with 0.17 mg g^−1^ DW and 0.18 mg g^−1^ DW. Other compounds found in the *V. myrtillus* cultures were quercetin and kaempferol derivatives and gallic acid derivatives. The UV chromatogram (*λ* = 280 nm) of the *V. myrtillus* culture Vm2 is presented in Fig. 3 alongside with a more detailed characterization of the compounds in Table 4.

**Table 4:** Compound identification for the *Vaccinium myrtillus* culture sample Vm2

Peak	Tentative compound identification	Molecular formula	MS^1^ (*m/z*)	MS^2^, characteristic fragments (*m/z*)^a^	Exact mass, observed (g mol^−1^)	Exact mass, calculated (g mol^−1^)	Error (ppm)
1	Coumaroyl hexose derivative	C_15_H_18_O_8_	651.1934 371.0981 325.0929	(371) 197.0446, 167.0337, 163.0387, 119.0481 (325) 163.0386, 119.0485			
2	Catechin	C_15_H_14_O_6_	869.2294 [3M−H]^−^ 579.1511 [2M−H]^−^ 289.0717 [M−H]^−^	(289) 245.0813, 125.0227	290.0784	290.0790	−2.0
3	Procyanidin tetramer (1 A-type bond)	C_60_H_48_O_24_	1151.2457 [M−H]^−^ 575.1200 [M−2H]^2−^	(1151) 407.0766, 289.0714, 285.0403, 245.0815, 125.0227	1152.2524	1152.2536	−1.1
4	Coumaroyl hexose	C_15_H_18_O_8_	651.1935 [2M−H]^−^ 325.0928 [M−H]^−^	(325) 163.0386, 119.0485	326.0995	326.1002	−2.1
5	Procyanidin dimer	C_30_H_26_O_12_	1155.2762 [2M−H]^−^ 577.1353 [M−H]^−^	(577) 407.0771, 289.0717, 245.0813, 125.0228	578.1420	578.1424	−0.7
6	Epicatechin	C_15_H_14_O_6_	869.2292 [3M−H]^−^ 579.1509 [2M−H]^−^ 289.0717 [M−H]^−^	(289) 245.0816, 125.0228	290.0784	290.0790	−2.0
7	Procyanidin pentamer (1 A-type bond)	C_75_H_60_O_30_	1439.3096 [M−H]^−^ 719.1517 [M−2H]^2−^	(719) 407.0764, 289.0715, 287.0560, 285.0403, 245.0454, 125.0228	1440.3163	1440.3169	−0.4
8	Procyanidin trimer (1 A-type bond)	C_45_H_36_O_18_	1727.3720 [2M−H]^−^ 863.1836 [M−H]^−^	(863) 407.0772, 289.0718, 287.0562, 285.0406, 245.0452, 125.0228	864.1903	864.1902	0.2
9	Procyanidin trimer (1 A-type bond)	C_45_H_36_O_18_	1727.3699 [2M−H]^−^ 863.1828 [M−H]^−^	(863) 407.0769, 289.0717, 285.0405, 245.0815, 125.0228	864.1895	864.1902	−0.8
10	Procyanidin tetramer	C_60_H_50_O_24_	1153.2596 [M−H]^−^ 576.1261 [M−2H]^2−^	(576) 407.0766, 289.0718, 287.0563, 285.0406, 245.0817, 125.0228	1154.2663	1154.2692	−2.5
11	Procyanidin pentamer	C_75_H_62_O_30_	1441.3235 [M−H]^−^ 720.1591 [M−2H]^2−^	(720) 407.0769, 289.0716, 287.0560, 285.0408, 245.0817, 125.0228	1442.3302	1442.3326	−1.7
12	Quercetin hexoside	C_21_H_20_O_12_	927.1843 [2M−H]^−^ 463.0884 [M−H]^−^	(463) 300.0272	464.0951	464.0955	−0.9
13	Procyanidin dimer	C_30_H_26_O_12_	577.1351 [M−H]^−^	(577) 407.0772, 289.0717, 245.0816, 125.0228	578.1418	578.1424	−1.0

Peak numbers correspond to those presented in Fig. 3

^a^The ions marked in parentheses were used for MS^2^ fragmentation experiments

The characteristic ions for the detected proanthocyanidins result from several types of cleavages; the ion at *m/z* 407 results from retro-Diels–Alder fragmentation and subsequent elimination of water (Friedrich et al. 2000), ions at *m/z* 289 and 287 result from quinone methide cleavage (Friedrich et al. 2000; Karonen et al. 2011), ion at *m/z* 245 likely from the loss of –CH_2_–CHOH group from a catechin unit (Pérez-Magariño et al. 1999), and ion at *m/z* 125 corresponds to phloroglucinol resulting from heterocyclic ring fission (Gu et al. 2003). The identities of peaks 4 and 1 are tentatively identified as coumaroyl hexose and a coumaroyl hexose derivative, respectively, due to their mass fragmentation patterns showing signals at *m/z* values 163 and 119 possibly resulting from coumaric acid and further cleavage of CO_2_ (Ma et al. 2007). Peak 12 was identified as a quercetin hexoside based on its UV spectrum and a product ion at *m/z* 300, resulting from the homolytic cleavage of the *O*-glycosidic bond of the hexose (Hvattum and Ekeberg 2003). In addition, a product ion at *m/z* 301 corresponding to the quercetin aglycone was detected in the UHPLC-DAD–3Q-MS^2^ analyses, confirming its identity.

Beside the sharp peaks listed in Table 4, a chromatographic hump is visible in the chromatogram in Fig. 3 between approximately 3 and 6 min. This corresponds to a mixture of a multitude of different isomers of proanthocyanidin oligo- and polymers, which are not resolved when using reversed-phase liquid chromatography, except for some small oligomers. The mean degree of polymerization for the proanthocyanidins in this hump, however, can be determined using MS^2^ methods for both procyanidins and prodelphinidins (Engström et al. 2014). For both of the two *V. myrtillus* culture samples, these mean degrees of polymerization were calculated to be 5.

The berries of *V. myrtillus* have long been known to be rich in anthocyanins (Suomalainen and Keränen 1961). They include five different anthocyanidin aglycones (cyanidin, delphinidin, peonidin, petunidin, and malvidin), all appearing with three glycones (arabinose, glucose, and galactose), giving it a characteristic fingerprint profile of 15 different anthocyanins, which has for example been suggested for use in *V. myrtillus* product authenticity studies (Primetta et al. 2013). The two studied *V. myrtillus* cell cultures differed from this profile slightly, as no malvidin aglycones were detected. All of the other four aglycones and their corresponding three types of glycosides were detected.

Besides anthocyanins, the phenolic compounds of *V. myrtillus* berries include other flavonoids, proanthocyanidins, hydroxycinnamic acids, and ellagic acid (Wildanger and Herrmann 1973; Törrönen et al. 1997; Häkkinen et al. 1999;).

The phenolic compounds of the leaves, similarly to berries, of *V. myrtillus* include anthocyanins, proanthocyanidins, flavonoids, hydroxycinnamic acids, coumaroyl iridoids, and cinchonains (Riihinen et al. 2008; Hokkanen et al. 2009). The leaf anthocyanins are present only in the red leaves of *V. myrtillus*, which result from exposure to sunlight and subsequent accumulation of anthocyanins to protect from UV-B radiation (Chalker-Scott 1999; Jaakola et al. 2004).

The polyphenol profile of the *V. vitis*-*idaea* L. culture was somewhat similar to that of the *V*. *myrtillus* culture, containing mostly procyanidins and quercetins, though especially procyanidins in much lower concentrations. The phenolic profile of the leaves of *V*. *vitis*-*idaea* has been determined to mostly consist of flavonoids, including catechin and epicatechin, and simple phenolic acids, with some proanthocyanidins, cinchonains, and coumaroyl iridoids (Ek et al. 2006; Hokkanen et al. 2009). The berries contain all of these, with the addition of anthocyanins (Andersen 1985; Häkkinen and Auriola 1998; Määttä-Riihinen et al. 2004a; Ek et al. 2006).

**Fig. 4:**
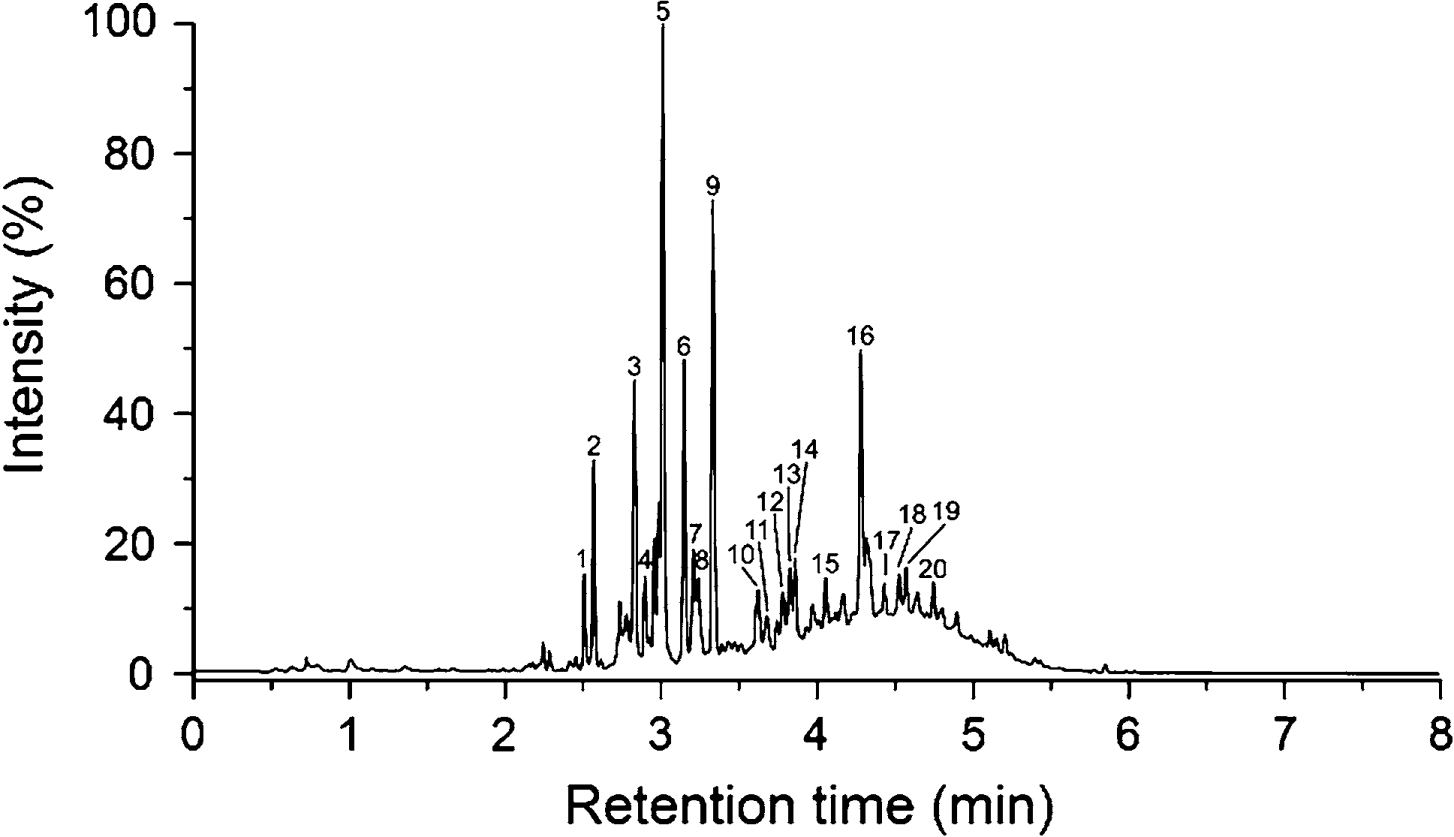
UHPLC-DAD chromatogram (*λ* = 280 nm) of the *E. nigrum* suspension culture sample En. For peak identification, see Table 5

The *E. nigrum* L. culture contained the highest concentration of prodelphinidins of all samples, with 2.03 mg g^−1^ DW. Its procyanidin content was higher than that (3.04 mg g^−1^ DW), but still distinctly less than the procyanidin content in *V. myrtillus*. Similar to *V. myrtillus*, the mean degree of polymerization of proanthocyanidins was calculated, and determined to be 2, showing clearly smaller oligomers compared to *V. myrtillus* samples on average. Anthocyanins with four different aglycones (cyanidin, delphinidin, peonidin, and petunidin) were detected as well. The UV chromatogram (*λ* = 280 nm) of the *E. nigrum* culture En is presented in Fig. 4 alongside with a more detailed characterization of the compounds in Table 5. Fragmentation patterns of the *E. nigrum* sample proanthocyanidins are very similar to the ones observed and described for *V. myrtillus*, and some ions are seen with 16 Da larger *m/z* values due to the additional hydroxylation of the B-ring in prodelphinidins compared to procyanidins. The coumaric acid derivatives and the quercetin hexose were similar as in *V. myrtillus*, and the caffeoyl hexoses (Roche et al. 2005) and naringenin hexoside (Sánchez-Rabaneda et al. 2004) were identified using the MS^2^ fragmentation data. Peak 14 remained unidentified.

**Table 5:** Compound identification for the *E. nigrum* culture sample En

Peak	Tentative compound identification	Molecular formula	MS^1^ (*m/z*)	MS^2^, characteristic fragments (*m/z*)^a^	Exact mass, observed (g mol^−1^)	Exact mass, calculated (g mol^−1^)	Error (ppm)
1	Caffeoyl hexose	C_15_H_18_O_9_	683.1831 [2M−H]^−^ 341.0874 [M−H]^−^	(341) 179.0337, 135.0435	342.0941	342.0951	−2.9
2	Coumaroyl hexose derivative		651.1934 371.0979 325.0929	(371) 163.0386, 119.0485 (325) 163.0386, 119.0485			
3	Caffeoyl hexose	C_15_H_18_O_9_	683.1831 [2M−H]^−^ 341.0870 [M−H]^−^	(683) 179.0337, 135.0435	342.0936	342.0951	−4.4
4	Catechin	C_15_H_14_O_6_	869.2296 [3M−H]^−^ 579.1511 [2M−H]^−^ 289.0718 [M−H]^−^	(289) 245.0814, 125.0227	290.0785	290.0790	−1.7
5	Coumaroyl hexose	C_15_H_18_O_8_	651.1934 [2M−H]^−^ 325.0919 [M−H]^−^	(325) 163.0386, 119.0485	326.0986	326.1002	−4.9
6	Coumaroyl hexose	C_15_H_18_O_8_	651.1934 [2M−H]^−^ 325.0917 [M−H]^−^	(325) 163.0386, 145.0279, 119.0484	326.0984	326.1002	−5.5
7	Caffeoyl hexose	C_15_H_18_O_9_	683.1824 [2M−H]^−^ 341.0870 [M−H]^−^	(683) 179.0337, 135.0435	342.0936	342.0951	−4.4
8	Procyanidin dimer	C_30_H_26_O_12_	1155.2764 [2M−H]^−^ 577.1353 [M−H]^−^	(577) 407.0770, 289.0716, 245.0818, 125.0228	578.1420	578.1424	−0.7
9	Epicatechin	C_15_H_14_O_6_	869.2303 [3M−H]^−^ 579.1510 [2M−H]^−^ 289.0718 [M−H]^−^	(289) 245.0812, 125.0227	290.0785	290.0790	−1.7
10	Procyanidin trimer (1 A-type bond)	C_45_H_36_O_18_	863.1832 [M−H]^−^	(863) 407.0767, 289.0715, 287.0559, 285.0403, 245.0450, 125.0228	864.1899	864.1902	−0.4
11	Procyanidin-prodelphinidin trimer (1 A-type bond)	C_45_H_36_O_19_	879.1785 [M−H]^−^	(879) 407.0770, 301.0352, 289.0717, 285.0404, 125.0228	880.1852	880.1851	0.2
12	Procyanidin-prodelphinidin dimer (A-type bond)	C_30_H_24_O_13_	1183.2353 [2M−H]^−^ 591.1150 [M−H]^−^	(591) 407.0768, 305.0662, 301.0352, 289.0713, 285.0404, 125.0227	592.1217	592.1217	0.1
13	Procyanidin trimer (1 A-type bond)	C_45_H_36_O_18_	863.1829 [M−H]^−^	(863) 407.0769, 289.0717, 285.0406, 125.0228	864.1896	864.1902	−0.7
14	Unknown		931.2152 465.1035	(465) 437.1089, 275.0561, 259.0612, 125.0228			
15	Quercetin hexoside	C_21_H_20_O_12_	927.1840 [2M−H]^−^ 463.0884 [M−H]^−^	(463) 300.0273	464.0951	464.0955	−0.9
16	Procyanidin tetramer (1 A-type bond)	C_60_H_48_O_24_	1151.2463 [M−H]^−^ 575.1198 [M−2H]^2−^	(575) 407.0768, 289.0716, 285.0404, 125.0228	1152.2530	1152.2536	−0.5
17	Procyanidin trimer (1 A-type bond)	C_45_H_36_O_18_	863.1835 [M−H]^−^	(863) 407.0768, 289.0715, 285.0402, 125.0227	864.1902	864.1902	0.1
18	Procyanidin dimer (A-type bond)	C_30_H_24_O_12_	1151.2451 [2M−H]^−^ 575.1201 [M−H]^−^	(575) 407.0769, 289.0714, 285.0402, 125.0227	576.1268	576.1268	0.1
19	Procyanidin trimer (2 A-type bonds)	C_45_H_34_O_18_	861.1673 [M−H]^−^	(861) 407.0769, 289.0715, 285.0402, 125.0228	862.1742	862.1745	−0.4
20	Naringenin hexoside	C_21_H_22_O_10_	433.1141 [M−H]^−^	(433) 271.0610, 151.0022	434.1208	434.1213	−1.1

Peak numbers correspond to those presented in Fig. 4

^a^The ions marked in parentheses were used for MS^2^ fragmentation experiments

The lipophilic phenolic extracts of *E*. *nigrum* leaves have been detected to contain chalcones, dihydrochalcones, and dihydrophenanthrene derivatives (Wollenweber et al. 1992), with the hydrophilic phenolic compounds of berries including hydroxycinnamic acids, flavonoids, proanthocyanidins, and high amounts of a wide range of anthocyanins (Määttä-Riihinen et al. 2004a).

The only suspension cultures that could produce proanthocyanidins in amounts comparable to that detected in natural plants were all from the family Ericaceae. In addition, all Ericaceae cultures were able to produce at least some amounts of flavonoids similar to their natural counterparts, as evidenced by the presence of kaempferol and quercetin derivatives.

As for the ratio of procyanidins to prodelphinidins, the detected 40:60 ratio of procyanidins to prodelphinidins in the *E*. *nigrum* culture is close to the ratio reported in *E*. *nigrum* berries (Määttä-Riihinen et al. 2004a). Furthermore, it is clearly different from the ratios in genus *Vaccinium* cultures, the proanthocyanidins of which consist almost exclusively of procyanidins.

### Families Caprifoliaceae and Poaceae

The *L. caerulea* L. var. *kamtschatica* (family Caprifoliaceae) culture was exceptional in that it contained by far the largest concentration of quinic acid derivatives in all of the samples, with only one other sample containing any at all. Quinic acid derivatives also contributed to the majority of its polyphenol profile, with trace amounts of gallic acid derivatives and procyanidins detected.

The taxonomy of some *Lonicera* species and the varieties of *L. caerulea* is not completely settled, and therefore, also the literature on *L. caerulea* var. *kamtschatica* is at times ambiguous. The berries contain high quantities of anthocyanins, along with other flavonoids, proanthocyanidins, and phenolic acids (Terahara et al. 1993; Chaovanalikit et al. 2004; Jurikova et al. 2011), while the leaves contain flavonoids and phenolic acids (Oszmianski et al. 2011).

Both the two included cereal cultures from family Poaceae, *A. sativa* L. and *H. vulgare* L. were low in the analyzed polyphenols, with trace amounts of gallic acid derivatives and procyanidins in both and trace amounts of quinic acid derivatives in one of the *H. vulgare* cultures. The phenolic content of *A*. *sativa* has been reported to consist mostly of other types of phenolics, mainly avenanthramides (Collins 1989), which are phenolic alkaloids, and simple phenolic acids (Durkee and Thivierge 1977). *H*. *vulgare* has also been studied for its phenolic content, and proanthocyanidins and flavonoids contribute to the majority of the phenolic compounds not bound to the cell wall (McMurrough et al. 1996; Ferreres et al. 2009).

### Culture conditions

As described, the suspension cultures used in this study were mostly grown under similar conditions, with the exception of the Poaceae samples As and Hv1. The growth media, however, differed between the samples (Table 1), with a total of six different media being used, which was due to the fact that the conditions were not optimized for polyphenol accumulation, but for general growth. The cultivation of the plant cells also differed for some samples as has been described earlier; most were cultivated in Erlenmeyer flasks, but different cultivation methods were used for Rc1, Rc2, Sa1, and Sa2. The accumulation of phenolic compounds in cell cultures is highly dependent on the concentrations of the plant growth regulators, auxins, and cytokinins, and their ratio (Dias et al. 2016). As for polyphenolic compounds, other published examples of the effects of these types of adjustments to the conditions include the inhibition of the production of ellagitannins by NH_4_^+^ in the medium (Ishimaru and Shimomura 1991) and the accumulation of anthocyanins and proanthocyanidins when using appropriate concentrations of sucrose (Decendit and Mérillon 1996). It must be noted that all these choices along with, for instance, the age of the culture and sub-culturing can cause the polyphenol profile to be vastly different even within a species. All of these naturally also influence how closely the cell cultures resemble their wild counterparts. Depending on these factors, reports on the polyphenolic profile of cell cultures may closely match that of the wild plants’ certain plant parts (e.g., our samples Vm1 and Vm2, Decendit and Mérillon 1996), be somewhat similar (e.g., our samples Rs1 and Rs2, López Arnaldos et al. 2001), or even remarkably different (e.g., our samples Sa1 and Sa2, Nohynek et al. 2014), making comparisons between different studies sometimes troublesome. Therefore, our results are not fully comparable in all cases to each other or to other culture studies involving the same species.

## Conclusions

Many of the studied cell suspension cultures proved to be relatively low in polyphenol content when compared to plants. The most distinctive exceptions to this were *S*. *aucuparia*, *V. myrtillus*, and *E. nigrum*, which, when combined, produced a comprehensive set of different polyphenolic compounds, including oligomeric tannins.

The cultures of the two families which were represented by three or more species showed capability to produce polyphenolic content representative for their natural counterparts. Rosaceae cultures produced the highest concentrations of hydrolysable tannins but not much proanthocyanidins, while Ericaceae cultures produced high concentrations of proanthocyanidins and in the case of *V. myrtillus* levels comparable to even its berries and leaves. Anthocyanins were detected in several of the species they naturally occur in, but the concentrations were lower than what has been detected in the berries.

Several cell suspension cultures originating from Rosaceae plants could be potentially used for further studies involving hydrolysable tannins, as some of them exhibited ellagitannin accumulation, and most of them produced galloyl glucoses.

### *Author contribution statement*

JS did the mass spectrometric analyses for all compound groups except for anthocyanins, analyzed the data, and wrote the bulk of the article. LN, HR, and RP-P designed and cultivated the cell cultures, and LN wrote the corresponding text. TS-L did the anthocyanin analyses and wrote the basis for the corresponding text. HR, J-PS, LN, and RP-P designed the research. All authors read, commented on, and approved the manuscript.

## Abbreviations

3Q: Triple quadrupole
DAD: Photodiode array detector
ESI: Electrospray ionization
MS^2^: Tandem mass spectrometry
U(H)PLC: Ultra-high-performance liquid chromatography
